# Exploring Adipokines in Assessing the Role of Dehydroepiandrosterone in Polycystic Ovary Syndrome-Linked Infertility

**DOI:** 10.7759/cureus.86921

**Published:** 2025-06-28

**Authors:** Sana Danish, Erum Gul, Shama Chaudhry, Madeeha Minhas

**Affiliations:** 1 Obstetrics and Gynaecology, Shaikh Zayed Hospital, Lahore, PAK; 2 Obstetrics and Gynaecology, Ziauddin University, Karachi, PAK; 3 Pathology, King Saud Bin Abdulaziz University for Health Sciences, Jeddah, SAU

**Keywords:** adipokines, adiponectin, dehydroepiandrosterone, leptin, polycystic ovary syndrome

## Abstract

Background: Polycystic ovary syndrome (PCOS) is an endocrine condition that affects women and results in imbalance prevalence, as well as infertility in most cases. Adipose tissue hormones, such as adiponectin and leptin, participate in metabolism and may influence the maturation of reproductive hormones. Dehydroepiandrosterone (DHEA) is a natural steroid that performs several functions, including fertility-enhancing effects. This can be attributed to the fact that it regulates hormones.

Objective: The objective of this research article is to note the effects that DHEA supplementation caused on reproductive hormones and adipokines in infertile women suffering from PCOS.

Methodology: A sample of 60 infertile women with PCOS desiring children was distributed into study groups, and the groups were classified at random and did not vary in terms of size. The researchers assigned Group A a 75 mg per day oral supplement of DHEA within 12 weeks, whereas Group B received no supplement at all. The hormones anti-Müllerian hormone (AMH), luteinizing hormone (LH), and estradiol (E2) were measured at baseline and at the end of the 12-week period. The level of leptin and adiponectin was also determined.

Results: The 12-week DHEA treatment resulted in increased AMH and estradiol levels when compared to the control group. Moreover, individuals within the DHEA group exhibited an increasing adiponectin level and a decreasing leptin level, which is a prediction of better metabolic efficiency.

Conclusion: The effect of DHEA on reproductive hormones and adipokine levels could be positive in women who have such benefits regarding infertility. The researchers conclude that the outcome of the study indicates that when these individuals are provided with DHEA, it enhances the probability of having a child. Nonetheless, it is essential to continue examining its safety and effectiveness over an extended period.

## Introduction

Polycystic ovary syndrome (PCOS) is one of the most common endocrine diseases affecting women of reproductive age, with an incidence of approximately 6% to 15% in women from their teenage years to menopause [1]. PCOS typically exhibits elevated amounts of male hormones, aberrant discharge of eggs, and ovarian cysts; it brings about sterility and is related to issues like insulin opposition, excess body fat, and unusual blood fats [2]. Since metabolic and reproductive problems are present in PCOS, there is a need to make an effort to find earlier drugs and markers that can treat both simultaneously [3]. On the same note, adipokines are bioactive peptides derived from adipose tissue that aid in regulating metabolic alterations, blood sugar levels, and inflammation in the body, which contributes to PCOS development [4].

Leptin and adiponectin have been a particular focus of research over the last few years. Leptin, which aids in regulating energy and reproduction, is often elevated in PCOS and is frequently associated with leptin resistance. Conversely, the amount of adiponectin is generally low among women with PCOS, but it effectively sustains better insulin use and has anti-inflammatory effects [5]. The change in adipokines may be accompanied by alterations in hormone levels and metabolism, potentially leading to reproductive complications in PCOS [6].

Dehydroepiandrosterone (DHEA), a body product that aids in the production of androgens and estrogens, has also been investigated in the context of reproductive biology [7]. Although it has shown excellent results in the treatment of patients with poor ovarian response, the effect that it has on hormones as well as adipokines in PCOS-related infertility remains obscure. Due to the multiple features of PCOS, combining the assessment of DHEA and adipokines can lead to the identification of new and effective methods of treating patients [8].

The purpose of this study is to investigate the impact of DHEA supplements on reproductive hormones and the primary adipokines, leptin and adiponectin, in women with PCOS-related infertility, to determine whether these changes may lead to improved fertility.

## Materials and methods

A prospective, controlled interventional study was carried out for 16 weeks, from December 2024 to April 2025, via non-probability consecutive sampling, including 60 infertile women with PCOS. The study obtained approval from the Ethical Review Committee of Ziauddin University, Karachi, and the Federal PGMI (approval no. 24-4136SZGY). The sample size was calculated using OpenEpi version 3.0.0 (released 2013, Atlanta, GA, USA). PCOS was officially diagnosed by meeting at least two of these Rotterdam criteria (2003): having oligo/anovulation, hyperandrogenism (visible or detected by tests), and polycystic ovaries on ultrasound [9].

Each group comprised an equal number of individuals who were assigned randomly. Group A (intervention group) received oral DHEA supplements (75 mg/day) for 12 weeks, whereas Group B (control group) received no treatment. Those who participated were between 20 and 38 years of age and had experienced infertility for more than 12 months without reproductive or endocrine problems. The inclusion criteria were definite PCOS and infertility, normal levels of prolactin and thyroid, and no use of hormones in the recent past. Exclusion criteria of this study comprised patients with adrenal hyperplasia, hyperprolactinemia, thyroid problems, uncontrolled diabetes, and those prescribed insulin-sensitizing drugs or corticosteroids. Baseline data were collected, which included a person's age, BMI, duration of infertility, and menstrual history. Blood samples were obtained at Day 0 and Week 12. Anti-Müllerian hormone (AMH), luteinizing hormone (LH), and estradiol (E2) were measured in serum by using commercial ELISA (enzyme-linked immunosorbent assay) kits purchased from BioTechne (Minneapolis, MN, USA) (qK7634 for AMH) and DRG Instruments (Marburg, Germany) (EIA-1780 for LH and EIA-2693 for E2). Moreover, the levels of leptin and adiponectin were determined using sandwich ELISA kits supplied by Sigma Aldrich (St. Louis, MO, USA) (RAB0333 for leptin; RAB1111 for adiponectin). Samples of blood were centrifuged at 3000 rotations per minute for 10 minutes and then preserved in a −80°C storage chamber until testing.

Both samples and assays in the laboratory were handled according to standard operating protocols (SOPs). Internal quality check methods were used to confirm the results from the ELISA. The data were first entered into Microsoft Excel (Microsoft Corporation, Redmond, Washington) and then analyzed using IBM SPSS Statistics for Windows, Version 26 (Released 2019; IBM Corp., Armonk, New York). The numeric values for quantitative variables were reported as means with corresponding standard deviations. Within the groups, independent t-tests were used to identify differences in means, and one-way ANOVA and chi-square tests were employed for variables involving more than two groups (p-value < 0.05, significant).

## Results

The study comprised 60 infertile women with PCOS, who were evenly divided into treatment (DHEA) and control groups. DHEA supplementation for 12 weeks resulted in noticeable enhancements in the levels of reproductive hormones and adipokines in the treatment group. There were no significant differences between the two groups in baseline characteristics. Table 1 presents the characteristics of the study participants, categorized by both age and medical history.

**Table 1:** Participant Demographics (n = 60) Chi-square and independent t-tests were used; p < 0.05 was considered statistically significant. BMI: body mass index; t-test: Student's t-test; χ²: chi-square; p-value: probability value; n: sample size; DHEA: dehydroepiandrosterone

Variable	DHEA Group (n = 30)	Control Group (n = 30)	Test Used	Test Value	p-value
Age (years)	28.4 ± 3.2	28.7 ± 3.0	t-test	t = 0.41	0.68
BMI (kg/m²)	26.9 ± 2.1	27.1 ± 2.3	t-test	t = 0.34	0.74
Duration of infertility (years)	3.1 ± 1.2	3.2 ± 1.1	t-test	t = 0.23	0.82
Menstrual irregularity (%)	83.3	86.6	Chi-square	χ² = 0.15	0.69

After intervention over a 12-week period, the group treated with DHEA had significantly higher levels of serum AMH and estradiol than the placebo group (p-values of 0.03 and 0.02, respectively). Additionally, the group that received DHEA had lower levels of LH (p = 0.01). Table 2 presents the differences in hormone levels observed after treatment.

**Table 2:** Hormonal Changes After 12 Weeks An independent t-test was used; p < 0.05 was considered statistically significant. AMH: anti-Müllerian hormone; LH: luteinizing hormone; pg/mL: picograms per milliliter; ng/mL: nanograms per milliliter; mIU/mL: milli-international units per milliliter; p-value: probability value

Hormone	DHEA Group (Post)	Control Group (Post)	Reference Range	Test Used	Test Value	p-value
AMH (ng/mL)	4.2 ± 0.8	3.5 ± 0.9	1.0–4.0 ng/mL	t-test	t = 2.24	0.03
LH (mIU/mL)	6.1 ± 1.0	7.5 ± 1.3	Follicular phase: 1.9–12.5 mIU/mL; ovulation peak: 8.7–76.3 mIU/mL; luteal phase: 0.5–16.9 mIU/mL	t-test	t = 3.45	0.01
Estradiol (pg/mL)	145 ± 25	120 ± 22	Follicular phase: 15-350 pg/mL; ovulation peak: 150-750 pg/mL; luteal phase: 30-450 pg/mL	t-test	t = 2.46	0.02

At the end of 12 weeks of DHEA treatment, findings showed that leptin decreased and adiponectin increased in the intervention group more than at baseline and even when compared to the control group (p = 0.01 for leptin and p = 0.02 for adiponectin), as shown in Table 3.

**Table 3:** Adipokine Levels Before and After Intervention An ANOVA test was used; p < 0.05 was considered statistically significant. ANOVA: analysis of variance; F: F-statistic (used in ANOVA); ng/mL: nanograms per milliliter; µg/mL: micrograms per milliliter; p-value: probability value

Adipokine	Pre-DHEA	Post-DHEA	Control (12 Weeks)	Reference Range	Test Used	Test Value	p-value
Leptin (ng/mL)	22.5 ± 2.3	18.1 ± 2.0	22.2 ± 2.5	4–25 ng/mL (Female)	ANOVA	F = 5.87	0.01
Adiponectin (µg/mL)	6.2 ± 0.9	8.4 ± 1.0	6.3 ± 1.1	5–30 µg/mL (Female)	ANOVA	F = 4.92	0.02

## Discussion

The use of DHEA (75 mg/day) for 12 weeks in patients with PCOS brought about significant changes in hormones and led to positive improvements in adipokines. These DHEA group participants presented with higher AMH levels (p = 0.03), higher estradiol levels (p = 0.02), and lower LH levels (p = 0.01). Leptin was seen to decrease from a level of 22.5 ± 2.3 ng/mL in the first visit to 18.1 ± 2.0 ng/mL in the second visit (p = 0.01); meanwhile, adiponectin increased from 6.2 ± 0.9 µg/mL to 8.4 ± 1.0 µg/mL (p = 0.02). DHEA is a steroid precursor that helps increase ovarian androgen levels while also enhancing FSH (follicle-stimulating hormone) receptor activity in the ovary, which in turn boosts AMH and estradiol levels [10,11]. Furthermore, DHEA may assist in adipokine signaling by helping to regulate insulin sensitivity and reducing low-grade chronic inflammation [12]. A decrease in leptin and an increase in adiponectin may indicate improved adipose tissue function, which is associated with better ovarian function and reproductive ability in PCOS. Performing these therapies together may benefit folliculogenesis and aid in restoring fertility [13].

Improvements in AMH, estradiol, and LH are aligned with studies done in DHEA-treated women. Gałczyńska et al. (2025) found that AMH and estradiol improved in patients with low ovarian reserve following DHEA supplementation. Additionally, reproductive guidelines suggest that AMH is an accurate method for measuring the number of small follicles in PCOS [14]. Our study highlights that changes in hormone levels take place in people with PCOS, suggesting that DHEA could aid in increasing the ovaries' reserve and maintaining balance in hormone levels in various fertility-related diseases [15]. Metabolic irregularities in PCOS are also indicated by changes in leptin and adiponectin levels. According to Merza et al. (2025), PCOS patients exhibited an increased value for leptin and a decreased value for adiponectin, indicating that leptin was associated with insulin resistance, while adiponectin was associated with better control of blood sugar levels [16].

Recently, Pratama et al. (2024) demonstrated that the LH/FSH and leptin/adiponectin ratios in women with PCOS are negatively correlated with each other [17]. From our interventions, it appears that DHEA may reduce leptin and increase adiponectin, which could lead to improved insulin sensitivity. Nikolettos et al. (2024) demonstrated that leptin and adiponectin are involved in regulating reproductive processes, in addition to influencing metabolism. Leptin at high levels can harm ovulation and the receptivity of the uterus, yet adiponectin is involved in the growth of follicles and the process of producing estrogen [18].

Certain limitations must be recognized. Because the number of participants (n=60) was low and the study lasted only 12 weeks, it is hard to reach long-term conclusions on clinical fertility issues, for example, ovulation or pregnancy. The study did not examine insulin resistance (for example, the homeostatic model assessment for insulin resistance (HOMA-IR)), perform biopsies of fat tissue, or explore ovarian genes, all of which could help explain the mechanisms driving the results. Further randomized controlled trials, preferably on larger cohorts, must follow these results and focus on fertility metrics. Additionally, future investigations could examine DHEA in conjunction with insulin-sensitizing medications.

## Conclusions

The study indicates that DHEA supplementation in women with PCOS and infertility leads to meaningful improvements in their reproductive hormone levels and certain adipokine levels. This suggests that DHEA may help improve fertility by regulating hormones and metabolic pathways.

These findings suggest that DHEA may play a role in regulating and balancing hormones and metabolism in PCOS. Nevertheless, extensive and continuous research is needed to confirm these findings and observe their effects on rates of ovulation and pregnancy.

## References

[1] Dar MA, Maqbool M, Ara I (2023). The PCOS puzzle: putting the pieces together for optimal care. Int J Adolesc Med Health.

[2] Dong J, Rees DA (2023). Polycystic ovary syndrome: pathophysiology and therapeutic opportunities. BMJ Med.

[3] Ibrahim SK, Alsaffar SF (2024). Assessment of monocyte chemoattractant protein-1 and fertility hormones in Iraqi women with polycystic ovarian syndrome. Ibn Al-Haitham J Pure Appl Sci.

[4] Naqvi I, Bandyopadhyay A, Panda A, Hareramadas B (2025). Polycystic ovarian syndrome: a review of multi-omics analyses. Reprod Sci.

[5] Baig M, Ahmad T, Tariq S (2024). Interplay of adipocytokines with polycystic ovary syndrome. Polycystic Ovary Syndrome.

[6] Xu Y, Zhu H, Li W, Chen D, Xu Y, Xu A, Ye D (2022). Targeting adipokines in polycystic ovary syndrome and related metabolic disorders: from experimental insights to clinical studies. Pharmacol Ther.

[7] Palomba S, Seminara G, Costanzi F, Caserta D, Aversa A (2024). Chemerin and polycystic ovary syndrome: a comprehensive review of its role as a biomarker and therapeutic target. Biomedicines.

[8] Kicińska AM, Maksym RB, Zabielska-Kaczorowska MA, Stachowska A, Babińska A (2023). Immunological and metabolic causes of infertility in polycystic ovary syndrome. Biomedicines.

[9] Smet ME, McLennan A (2018). Rotterdam criteria, the end. Australas J Ultrasound Med.

[10] Chen P, Jia R, Liu Y, Cao M, Zhou L, Zhao Z (2022). Progress of adipokines in the female reproductive system: a focus on polycystic ovary syndrome. Front Endocrinol (Lausanne).

[11] Schüler-Toprak S, Ortmann O, Buechler C, Treeck O (2022). The complex roles of adipokines in polycystic ovary syndrome and endometriosis. Biomedicines.

[12] Chen X, Jia X, Qiao J, Guan Y, Kang J (2013). Adipokines in reproductive function: a link between obesity and polycystic ovary syndrome. J Mol Endocrinol.

[13] Zuo M, Liao G, Zhang W (2021). Effects of exogenous adiponectin supplementation in early pregnant PCOS mice on the metabolic syndrome of adult female offspring. J Ovarian Res.

[14] Gałczyńska D, Daniluk J, Buczek-Kutermak A, Pruś P, Pluta D (2025). Decoding the relationship between polycystic ovary syndrome and hormonal dependencies of anti-Müllerian hormone and other markers. Biomedicines.

[15] Zhang Y, Yu X, Liu L, Zheng J (2023). Mechanisms of DHEA-induced AMH increase in the ovarian tissues of PCOS rat. Reprod Biol.

[16] Merza WM, Yaseen AK, Mahmood MA (2025). FSH, LH, lipid and adipokines in polycystic ovary syndrome: clinical biochemistry insights for diagnosis and management. J Steroid Biochem Mol Biol.

[17] Pratama G, Wiweko B, Widyahening IS (2024). Body composition parameters, adiponectin, leptin and adiponectin/leptin ratio are correlated with LH/FSH ratio in women with PCOS but not in women without PCOS. Indones J Obstet Gynecol.

[18] Nikolettos K, Vlahos N, Pagonopoulou O (2024). The association between leptin, adiponectin levels and the ovarian reserve in women of reproductive age. Front Endocrinol (Lausanne).

